# Adrenal carcinoma: a case report

**DOI:** 10.1186/s13256-022-03398-4

**Published:** 2022-05-30

**Authors:** D. R. Abeynayake, Sopan V, K. J. C. Perera, A. Paramanantham, T. M. J. Munasinghe

**Affiliations:** National Hospital of Sri Lanka, Colombo 10, Sri Lanka

**Keywords:** Adrenal carcinoma, Microscopic hematuria, Cushing disease, Endocrine hypertension

## Abstract

**Background:**

Adrenocortical carcinoma is a rare malignancy (0.5–2 cases/million/year) with a poor prognosis. Hypercortisolism, virilization, and compressive features are among the common presentations of adrenocortical carcinoma. Hematuria is one of the rare initial presentations of adrenocortical carcinoma reported in the literature. We report a case of adrenal carcinoma presenting with microscopic hematuria.

**Case presentation:**

A 67-year-old Sri Lankan patient with diabetes, hypertension, and ischemic heart disease presented with an acute coronary event. During the routine evaluation, microscopic hematuria was detected without proteinuria or active sediments. She denied any painful micturition, previous similar episodes, or abdominal pain. Further evaluation revealed a hypokalemia with biochemical evidence of hypercortisolism and high testosterone levels with suppressed adrenocorticotropic hormone levels. On imaging, there was evidence of a right suprarenal mass 7 cm × 3 cm × 6 cm in size that was hypoechoic and lobulated and suggestive of a lipid-poor tumor. She underwent adrenalectomy. By the time of surgery 3 weeks later, significant weight gain with features of Cushing syndrome, including hirsutism, skin atrophy, easy bruising without virilization, and proximal myopathy, were noted. Histology identified a right-sided adrenal tumor with capsular and vascular invasion. Hypercortisolism and hematuria disappeared after surgery. The patient was referred for further oncological management.

**Conclusion:**

This case illustrates a rare presentation of adrenal carcinoma. Awareness of this presentation may facilitate early evaluation and management.

## Background

Adrenocortical carcinoma (ACC) is a rare malignancy (0.5–2 cases/million/year) with a poor prognosis [1]. Hypercortisolism, virilization, and compressive features are among the common presentations of ACC. Forty-five percent (45%) of adrenocortical carcinomas present as Cushing syndrome, 25% as Cushing with virilization, and 10% with virilization alone [2] These tumors can invade surrounding vessels, resulting in poor prognosis. Infrequently, tumor thrombus can extend to the renal vein, inferior vena cava, and right atrium [1]. Hematuria is rarely reported in the literature as the initial presentation of the ACC [3]. We report a case of adrenal carcinoma presenting with microscopic hematuria.

## Case report

A 67-year-old Sri Lankan lady with a history of diabetes and hypertension and ischemic heart disease presented to the National Hospital of Sri Lanka (NHSL) with a history suggestive of ischemic chest pain. While she was being treated for an acute coronary event, microscopic hematuria with 50–60 red cells without proteinuria or casts was noted. She denied any painful episodes of micturition, abdominal pain, or previous similar episodes. Abdominal examination was unremarkable. Urine for dysmorphic cells was negative. She also had hypokalemia (serum potassium 3.1 mEq/l). Her cardiac function was satisfactory. Renal, liver, and thyroid functions were normal. Ultrasound scan revealed a right-sided, 7 cm × 3 cm × 6 cm, suprarenal mass that was hypoechoic and lobulated. Contrast-enhanced computed tomography (CT) scan of abdomen was done and revealed a well-defined oval-shaped, soft-tissue-density lesion in the right suprarenal region suggestive of a lipid-poor adenoma. Her 9 am cortisol was 628 (166–507) nmol/l, serum free testosterone was 14.3 (0.1–1.7) pg/ml, and dehydroepiandrosterone (DHES) was 3.91 (0.03–5.8) μg/ml. Cortisol was not suppressed with high-dose dexamethasone. Adrenocorticotropic hormone (ACTH) level was < 5 (10–50) pg/ml. Renin–aldosterone ratio and urine vanillylmandelic acid were normal. While awaiting surgery for adrenal carcinoma, in 3 weeks’ time, significant weight gain was noted with an increase in body mass index (BMI) from 26.5 to 30 kg/m^2^. Gradually she developed hirsutism, thin skin, and easy bruising. However, there were no features of virilization or proximal muscle weakness. During the investigation, she developed hypertensive heart failure with recurrent non-ST-elevation myocardial infarctions, which was treated in the intensive care unit. With greatest precautions, adrenalectomy was performed. Histology revealed right-sided adrenal carcinoma with vascular and capsular invasion. Following the surgery, her cushinoid features were abated. Latest 9 am cortisol was 485 (166–507) nmol/l, serum free testosterone was 1.1(0.1–1.7) pg/ml, and low-dose dexamethasone suppression test was suppressed to 46 (50) nmol/l. Hematuria settled. She was referred for further specialized oncological care with the histology report.

**Figure:**
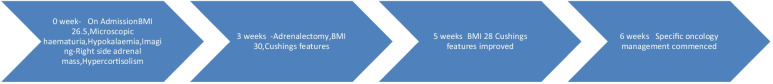

## Discussion

Adrenocortical carcinoma (ACC) is a rare malignancy with an incidence of 0.5–2 cases/million/year [1]. ACC can occur at any age, with a peak incidence between 40 and 60 years and women being more often affected (55–60%) [2]. ACC may present as a functionally active adrenal gland in about 50–60% of patients. Hypercortisolism (Cushing syndrome) or mixed Cushing and virilizing syndromes are observed in the majority of these patients. Compressive symptoms such as abdominal discomfort, vomiting, and back pain may be present in 30–40%. In 10–15% of patients, the diagnosis is an incidental finding. Constitutional symptoms are rarely present [2]. Unusual clinical manifestations reported include hematuria, lower-extremity edema, Budd–Chiari syndrome, acute abdominal pain, pelvic pain, urinary obstruction, and paraplegia [4]. Early diagnosis is vital owing to its poor overall prognosis. Five-year survival is 60–80% for tumors confined to the adrenal space, 35–50% for locally advanced disease, and much lower in case of metastatic disease with reported percentages ranging from 0% to 28% [2]. It is critical to know whether the adrenal mass is malignant or not. Suspicion of ACC might arise if the patients rapidly develop features of adrenocortical hormone excess such as weakness, hypokalemia, wasting and constitutional symptoms, or features of a large abdominal mass. Hirsutism or virilization in women or recent onset gynecomastia in men might be due to excess sex hormone production by an ACC. Evidence of co-secretion of different steroids raises suspicion of ACC (especially if sex hormones are involved). ACCs are usually large (> 4 cm) and of inhomogeneous appearance, and characterized by low fat content [2]. Our patient had a lipid-poor tumor larger than 4 cm, though she did not have features of local invasion on imaging. However, definite differentiation between adenoma and carcinoma should be done after pathological assessment.

Advanced adrenal carcinomas present with vascular invasion mainly into renal vein and inferior vena cava (IVC). Since this patient had histologically proven vascular invasion, this is the most likely explanation for the hematuria. Right-sided ACC may grow into the IVC more frequently than left-sided tumors. Left-sided ACC extends into the IVC through the renal vein [1]. There is a similar presentation of right-sided ACC without radiological evidence of local invasion reported in the literature [5].

However, hematuria being the initial presentation of the ACC before the onset of features of hyper functioning adrenal gland is an unusual presentation.

Definitive cure requires adrenalectomy with intra- and postoperative glucocorticoid replacement in all patients with autonomous cortisol secretion followed by mitotane treatment if indicated [2]. To date, mitotane has been the only drug that has proven effective in treating patients with metastatic adrenocortical carcinoma [4]. Adjuvant mitotane treatment is recommended in patients without macroscopic residual tumor after surgery but who have a perceived high risk of recurrence [2].

## Conclusion

This case illustrates a rare presentation of a case of adrenal carcinoma. Our patient had a right-sided ACC with histologically proven local vascular invasion that may have caused the hematuria with possible micrometastasis. Since this is a rare condition with poor overall prognosis, awareness of this rare presentation may facilitate early evaluation and management.

## Data Availability

Not applicable.

## Abbreviations

ACC: Adrenocortical carcinoma
NHSL: National Hospital of Sri Lanka
ACTH: Adrenocorticotropic hormone
BMI: Body mass index
DHES: Dehydroepiandrosterone

## References

[1] Yadav R, Dassi V, Kumar A. Adrenocortical carcinoma with inferior vena cava thrombus: renal preserving surgery. Indian J Urol. 2016;32(2):161–3. 10.4103/0970-1591.174782. PMC4831509 27127363

[2] Fassnacht M, Dekkers OM, Else T, Baudin E, Berruti A, de Krijger R, Haak HR, Mihai R, Assie G, Terzolo M. European Society of Endocrinology Clinical Practice Guidelines on the management of adrenocortical carcinoma in adults, in collaboration with the European Network for the Study of Adrenal Tumors. Eur J Endocrinol. 2018;179(4):G1–46. 10.1530/EJE-18-0608. 30299884

[3] Wajchenberg BL, Albergaria Pereira MA, Medonca BB, Latronico AC, Campos Carneiro P, Alves VA, Zerbini MC, Liberman B, Carlos Gomes G, Kirschner MA. Adrenocortical carcinoma: clinical and laboratory observations. Cancer. 2000;88(4):711–36. 10679640

[4] Wajchenberg BL, Albergaria Pereira MA, Medonca BB, Latronico AC, Carneiro PC, Ferreira Alves VA, Zerbini MCN, Liberman B, Gomes GC, Kirschner MA. Adrenocortical carcinoma. Cancer. 2000;88:711–36. 10679640

[5] Dixon AN, Bing RF. Two cases of adrenocortical carcinoma presenting as Conn’s syndrome. J Hum Hypertens. 2001;15(1):75–9. 10.1038/sj.jhh.1001105. 11224006

